# Peripheral nerve implants enriched with chemotactic factors for peripheral nervous tissue engineering

**DOI:** 10.1186/2193-1801-4-S1-L30

**Published:** 2015-06-12

**Authors:** Katarzyna Nawrotek, Michał Tylman, Jacek Balcerzak, Kamil Kamiński

**Affiliations:** Lodz University of Technology, Poland

**Keywords:** Biomaterials, hydrogel, nerve regeneration

Peripheral nerve injuries are an important part of everyday medical practice since there are reported over 600,000 cases in Europe and in the United States annually [1]. Many of such nerve injures cause gaps between nerve stumps. Without intervention, they can lead to the formation of a stump neuroma, what can result in functional impairment of the nerve fiber. The current approaches to regeneration of damaged peripheral nerves include: autografting, allografting, and, last but not least, the implantation of polymeric tubes and conduits between nerve stumps. Nerve autografting is considered as the “gold standard” technique for the repair of peripheral nerve discontinuities, but it has a number of limitations, such as the requirement for the second surgical procedure to harvest the graft tissue, the donor site morbidity, additional injuries and scarring as well as the increased recovery time. Allografts (e.g., cadaver nerve grafts) and xenografts (e.g. animal nerve grafts) can be an alternative to autografts, but their main drawback lies in the high possibility of an undesirable immune response. The most promising materials for peripheral nerve conduits preparation are natural biopolymers. They are obtained from natural sources, exhibit similar properties to the tissues they are replacing and reveal good cell adhesion. Furthermore, they tend to accelerate regeneration processes due to specific chemical interactions within the human body, e.g. with extracellular matrix (ECM) molecules. The purpose of our study is to create a conduit with properties that will mimic the ones of the extracellular matrix of the peripheral nervous system. Our focus is put on natural polymers, especially chitosan. In addition, we use chemotactic factors which exhibit properties beneficial in regeneration of the peripheral nervous tissue.
